# Hybrid Harmony Search–Artificial Intelligence Models in Credit Scoring

**DOI:** 10.3390/e22090989

**Published:** 2020-09-04

**Authors:** Rui Ying Goh, Lai Soon Lee, Hsin-Vonn Seow, Kathiresan Gopal

**Affiliations:** 1 Laboratory of Computational Statistics and Operations Research, Institute for Mathematical Research, Universiti Putra Malaysia, Serdang, Selangor 43400, Malaysia; gr.ying@hotmail.com (R.Y.G.); kathiresan@upm.edu.my (K.G.); 2 Department of Mathematics, Faculty of Science, Universiti Putra Malaysia, Serdang, Selangor 43400, Malaysia; 3 Nottingham University Business School, University of Nottingham Malaysia, Semenyih, Selangor 43500, Malaysia; Hsin-Vonn.Seow@nottingham.edu.my

**Keywords:** credit scoring, support vector machines, random forest, harmony search, feature selection, hyperparameter tuning, artificial intelligence

## Abstract

Credit scoring is an important tool used by financial institutions to correctly identify defaulters and non-defaulters. Support Vector Machines (SVM) and Random Forest (RF) are the Artificial Intelligence techniques that have been attracting interest due to their flexibility to account for various data patterns. Both are black-box models which are sensitive to hyperparameter settings. Feature selection can be performed on SVM to enable explanation with the reduced features, whereas feature importance computed by RF can be used for model explanation. The benefits of accuracy and interpretation allow for significant improvement in the area of credit risk and credit scoring. This paper proposes the use of Harmony Search (HS), to form a hybrid HS-SVM to perform feature selection and hyperparameter tuning simultaneously, and a hybrid HS-RF to tune the hyperparameters. A Modified HS (MHS) is also proposed with the main objective to achieve comparable results as the standard HS with a shorter computational time. MHS consists of four main modifications in the standard HS: (i) Elitism selection during memory consideration instead of random selection, (ii) dynamic exploration and exploitation operators in place of the original static operators, (iii) a self-adjusted bandwidth operator, and (iv) inclusion of additional termination criteria to reach faster convergence. Along with parallel computing, MHS effectively reduces the computational time of the proposed hybrid models. The proposed hybrid models are compared with standard statistical models across three different datasets commonly used in credit scoring studies. The computational results show that MHS-RF is most robust in terms of model performance, model explainability and computational time.

## 1. Introduction

Credit risk evaluation is a crucial routine of risk management in financial institutions. Credit scoring models are the main tool utilized to make credit granting decisions where the probability of default resembles the entropy concept, i.e., probabilistic measure of uncertainty. Hence to better measure risk, more accurate classification models are needed. Though statistical models are usually the preferred option, Artificial Intelligence (AI) models are beginning to be favoured for their accuracy and flexibility in the face of the volume of data. Advances in these techniques have further increased their popularity particularly in risk assessments. Support Vector Machines (SVM) and Random Forest (RF) are the main AI classifiers used in this study as recommended in two large scale benchmark studies by [1,2], respectively, due to their competitive performance as compared to other classifiers. There are two issues to be considered when using SVM and RF, i.e., sensitivity to hyperparameters settings and the black-box property.

For hyperparameter tuning, Grid Search (GS) has always been the conventional tuning tool for both SVM and RF. Recently, the metaheuristic approaches (MA) have shown potential as a competitive tool to tune SVM hyperparameters [3]. Some works utilized Genetic Algorithm (GA) [4,5] and Particle Swarm Optimization (PSO) to tune SVM [6,7]. A recent work experimented with MA [8] by using Artificial Bee Colony (ABC). These MA techniques have reported competitive results, indicating the potential of MA to be used to tune SVM hyperparameters.

For RF hyperparameter tuning, the major approach is the repeated trial-and-error tuning which requires subjective judgement from researchers [9,10,11,12,13]. Some researches tune the hyperparameters by examining a certain input range which is available in some software toolbox [2,14]. The GS is still a popular technique to tune RF [15,16]. Reference [16] compared GS with Random Search and PSO, and pointed out the benefit of PSO. Despite manual tuning being the common approach, experiments with PSO [16] shows the potential of MA.

Solving the black-box property is a challenging task. For SVM, feature selection strategy to enable explanation on reduced features is frequently attempted. GA has shown its potential in developing a different wrapper GA-SVM with the ability to reduce the features of SVM, yet maintaining a good model performance. References [17,18] incorporated information from a filter technique as the input to the GA wrapper. References [19,20] proposed a hybrid GA-SVM to perform hyperparameter tuning and feature selection simultaneously, whereas [20] included feature weighting in the wrapper GA-SVM model.

RF has the advantage of being able to explain the attributes with the computed feature importance. References [9,13] provided attributes information based on the feature importance. Reference [12] used this benefit for feature screening. Reference [10] incorporated the feature importance with profit measures while [11,21] built new credit scoring models with the feature ranking.

Despite being the most common technique for hyperparameter tuning, GS is a rigid brute force technique that will search through all possible combinations of the hyperparameters. For continuous hyperparameters, the computational effort will increase as the granularity of the search range increases. In addition, using GS to conduct feature selection will tremendously increase the computational time due to the increased features search space. Thus, to address both hyperparameter tuning and model explainability simultaneously, MA is a potential candidate tool to be hybridized with SVM and RF, with GA being the most commonly used method in the past. Recently, Harmony Search (HS) has received attention to be hybridised with SVM [22,23,24] and RF [25] in various domains for the purpose of hyperparameter tuning or feature selection. The authors of [26] have reviewed works using HS to conduct feature selection along with the use of different machine learning algorithms across various domains. Despite the successful implementations of HS for hyperparameter tuning and feature selection, ref. [27] has been the only study utilizing HS for feature selection in the nearest neighbourhood credit scoring model.

To the best of our knowledge, HS has yet to be hybridized with SVM and RF for the purpose of simultaneous hyperparameter tuning and model explainability in credit scoring. The HS first developed by [28] is inspired by the music improvisation process, where musicians tune their instruments’ pitch to achieve perfect harmony in seeking for an optimal solution. Hence, two hybrid models, i.e., HS-SVM and HS-RF are proposed in this study. HS-SVM conducts hyperparameter tuning and feature selection simultaneously to select appropriate hyperparameters and explain the attributes based on the reduced features. The HS-RF conducts hyperparameter tuning to ensure good model performance to provide reliable feature importance. SVM and RF are then hybridized with a modified HS (MHS) to improve the computational efficiency yet maintaining a comparable performance of the GS and HS. Parallel computation is applied on MHS hybrid models to improve the computational time. Then, the proposed models are compared with standard statistical models across two well-known credit scoring datasets and a peer-to-peer lending dataset. The discussions are based on model performances, model explainability, and computational time. Competitive results of the proposed models highlight the flexibility of utilizing HS as compared to GS, i.e., the ability to conduct feature selection and search for continuous hyperparameters without the need to specify the granularity of the search range. In addition, the competitiveness of MHS hybrid models further demonstrates the flexibility of HS to be modified to improve computational efficiency.

Research in credit scoring studies have been continuously attempted with various AI techniques. Several recent studies have marked a paradigm shift towards the usage of non-linear advanced techniques [29] and ensemble models [30,31,32] to be the potential techniques to achieve good performance in handling various types of data patterns. Due to the property of decision tree models being the de-facto community standard in classification task [33], tree-based ensembles have received attention in [31,32] with competitive performances reported on credit scoring datasets. In line with the recommendation from the large scale benchmark studies [1,2] and the paradigm shift observed in recent literatures, the proposed models in this study which are based on SVM and RF are aligned with the current trend of utilizing non-linear AI techniques and tree-based models. Most of the recent studies with the new advanced AI techniques have been a direct application of the models to assess their performance on credit scoring datasets to investigate their potential, with only [31] included synthetic features to address model explainability. Instead of a direct application for investigative purpose, the proposed hybrid models provide new idea to improve performance via simultaneous hyperparameters tuning and features selection. Besides, usage of RF has the benefit compared to the other tree-based ensembles because the computed features ranking is an appropriate tool for model explanation.

This paper is organized as follows. Section 2 provides an overview of the HS algorithm and the numerical experiments to demonstrate the potential of HS hybrid models, leading to the intuition to develop MHS hybrid models. Section 3 details on the hybrid models’ formulation. Section 4 elaborates on the experimental setup. Then, Section 5 reports the computational results with detailed discussions. Finally, Section 6 concludes the study and provides possible future directions.

## 2. Harmony Search to Modified Harmony Search

### 2.1. Harmony Search

The HS metaheuristic is a random search technique guided by fitness function evaluations. The HS search process is controlled by explorative and exploitative operators to seek solutions from the search range. A standard HS algorithm [28] consists of five procedures as follows:

1. Definition of objective function and the required parameters. The parameters are Harmony Memory Size (HMS), Harmony Memory Considering Rate (HMCR), Pitch-Adjustment Rate (PAR), bandwidth (bw), and maximum iterations (max_iter).
2. Initialization of Harmony Memory (HM). HM consists of the HMS number of possible candidate solutions with *n* dimensions which depends on the number of decision variables. All the candidate solutions in HM are generated from a uniform distribution that is based on the decision variables’ range.
3. Improvisation to generate new harmony. There are two main operators, i.e., HMCR to control the exploration and PAR to control the exploitation of the search process. HMCR is the probability of selecting a new harmony from HM, while its counterpart (1−HMCR) is the probability to randomly generates a new harmony. A low HMCR indicates high explorative power of the search process, because the search process will continuously generate a new harmony out of HM by exploring different search spaces. PAR is the probability to improvise the selected harmony from HM by moving to the neighbouring values with a step size of bw (for continuous variables) or one step to the left or right (for discrete variables). A low PAR indicates low exploitation power to conduct local exploitation of the harmony around its neighbourhood.
4. Update HM. The new harmony from (3) is evaluated against the fitness function. Replace the worst solution in HM with the new harmony if it has a better fitness value.
5. Termination. Repeat (3) and (4) until max_iter has reached.

### 2.2. Numerical Experiment Part I: Potential of HS Compared to GS

A numerical experiment is conducted to compare the performance of HS hybrids with the GS approach for hyperparameter tuning. For demonstration purposes, the numerical experiment is implemented on the German credit dataset and evaluated with the Area Under Receiver Operating Characteristics (AUC) from the average of 10-fold cross validation. Details of HS-SVM and HS-RF are enclosed in Section 3.1 and Section 3.2, while the details of the German dataset can be found in Section 4.1.

SVM hyperparameters search range follows the recommended settings by [8], i.e., $\log_{2}C=\left[-5,12\right]$ and $\log_{2}\gamma =\left[-12,5\right]$. Both hyperparameters are continuous variables that can take any values within the range. For GS, it is important to determine the granularity of the grid. Thus, GS for SVM will first search at a coarse grid of $\log_{2}C=\left[-5,-4,-3,\ldots ,11,12\right]$ and $\log_{2}\gamma =\left[-12,-11,-10,\ldots ,4,5\right]$ then follow with a finer grid of a granularity of 0.05 around the best returned solution from the coarse grid. For HS-SVM, the search process will examine the whole search range by picking the values via a uniform distribution, without the need to set the granularity.

RF hyperparameters are discrete variables with the search range of ntree={100,200,…,500} and mtry={1,2,…,a}, where *a* is the total number of attributes available. For both GS and HS, the search will be conducted across the search range but the granularity will not have to be determined as both are discrete variables.

In order to show that HS is a competent tool, the main parameters in HS, i.e., HMCR and PAR have to be robust across small perturbations. To set up for the experiment, a recommended range from [34] is applied (HMCR=[0.70,0.95], PAR=[0.10,0.35]) for both parameters. Both parameters are perturbed at a small change of 0.05 to examine their robustness. The AUC performances and computational time are reported in Table 1.

In terms of the model performance, both HS-SVM and HS-RF have reported a high mean AUC with only 0.2% and 0.1% standard deviation, respectively, across all the different combinations of HMCR and PAR. Despite the small perturbations of HMCR and PAR, HS-SVM and HS-RF still result in a stable performance, indicating HS is a robust tool to be hybridized with SVM and RF for hyperparameter tuning. Besides this, the results from Table 1 also imply that the HMCR and PAR range recommended by [34] is reliable as all the models have reported a competitive AUC across the whole range of the two operators.

However, the computational time deviation for HS-SVM and HS-RF are slightly higher. This is due to the effect of different hyperparameters that require different computational power. During the search process, HS may explore different areas of the hyperparameters search space followed with exploitation in the neighbourhood areas. Hence, depending on the search areas led by the operators, the different combinations of the operators will lead to different search areas where some consume more computing power than the others.

GS-tuned SVM and RF are compared with HS-SVM and HS-RF, respectively (see Table 2). HS hybrid models have achieved a slightly better model performance (higher AUC). HS-SVM is effective in the computational time and show competitive AUC performance. The extra computational effort for GS approach is due to the continuous search space of SVM hyperparameters that require the GS process to first search on a coarse grid followed by a finer grid. This is the main advantage of HS which is able to save computational effort by not needing to determine the granularity of the continuous search space. Due to the discrete hyperparameters search space for RF, there is no huge difference in the computation time between the GS-tuned RF and HS-RF.

The HS has demonstrated its potential as a competent tool to tune SVM and RF, with max_iter as the only stopping criteria. The competent AUC performance reported in Table 1 implies max_iter=100 is sufficient for the search. The bw assistant exploitation tool controls how far the new solution should be adjusted. HS-SVM has bw=0.1, which is an appropriate width to move around the SVM hyperparameters search range. Since HS-RF has discrete decision variables, settings of bw is not required.

Since the HS is more computationally efficient than the GS (depending on the max_iter), this inspired the idea to further enhance the computational efficiency by having the whole search process end with lesser number of iterations. However, it will be doubtful to set a low max_iter as there may still be more space to be searched upon. Therefore, the convergence pattern of the hybrid HS models have to be observed at different levels of exploration and exploitation.

### 2.3. Numerical Experiment Part II: Intuition to Develop MHS Hybrid Models

#### 2.3.1. Search Patterns of HS-SVM and HS-RF

**Figure 1:**
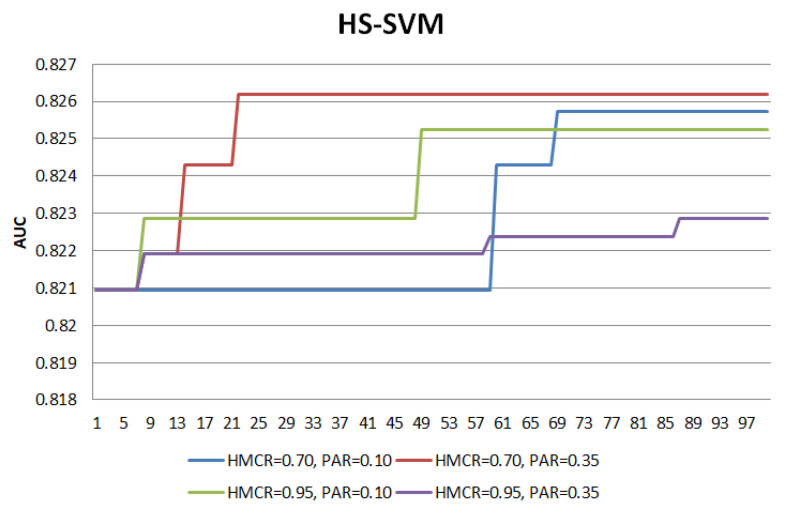
Search pattern of HS-SVM.

**Figure 2:**
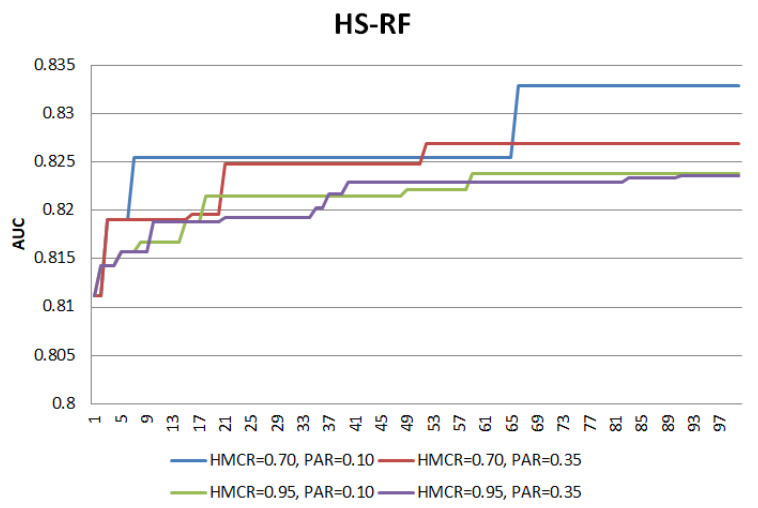
Search pattern of HS-RF.

Figure 1 and Figure 2 illustrate the search patterns for HS-SVM and HS-RF at four different combinations of HMCR and PAR operators for the first fold of the cross validation. HMCR=0.70 and HMCR=0.95 indicate higher and lower exploration, respectively, while PAR=0.10 and PAR=0.35 indicate for lower and higher exploitation, respectively. Generally, these combinations lead to only a slight difference in the final AUC performance. However, the search patterns of the different combinations show how exploration and exploitation control the search process. The search process is said to reach a convergence when ‘a plateau’ pattern is observed.

For both HS-SVM and HS-RF, the lower HMCR value has demonstrated higher explorative power compared to the higher HMCR value, given that PAR settings are constant (HMCR=0.70,PAR=0.10/HMCR=0.95,PAR=0.10 and HMCR=0.70,PAR=0.35/HMCR=0.95,PAR=0.35). At a lower HMCR value, the curves show sharp increment patterns, indicating active movement to different search areas. On the other hand, at a higher HMCR value, the increment patterns have lower gradient during the transition to higher AUC. The sharper transitions show that global search takes more dominant role than local search in the process.

As for the PAR operator, the lower PAR value has demonstrated lower exploitative power compared to the higher PAR value, given that HMCR settings are constant (PAR=0.10,HMCR=0.70/PAR=0.35,HMCR=0.70 and PAR=0.10,HMCR=0.95/PAR=0.35,HMCR=0.95). At a higher PAR value, the curves assist in the global search with more transition points before moving to another search space with a higher AUC. This indicates a more active local search at a higher PAR to improve the AUC by shifting to the neighbourhood search area.

Towards the end of the search process, ‘a plateau’ pattern is observed, indicating the process has reached convergence. Overall, all the different combinations have reached convergence, with certain combinations showing earlier convergence and the others. Thus, the main intuition for the development of a modified HS (MHS) is to achieve a comparable performance as the HS but at an earlier convergence to save up extra computational effort. The main modifications on HS to develop a MHS are as follows:

1. Elitism selection during memory consideration
  The selection of new harmony is no longer a random selection from HM, but with an objective to select a better quality harmony. Elitism selection leads the search process to focus on better quality candidates, thus enabling a faster convergence. Harmony vectors in HM are divided into two groups, i.e., elite (g1) and non-elite (g2), where g1 consists of harmony vectors with better performance than g2.
  Each harmony vector in HM takes an index number from the sequence of {1,HMS}. Since HM is sorted in the order of best to worst performance, harmony vectors with lower index number indicate their potential as the candidates in the elite group. The first quartile, q1 of the index sequence is computed as in Equation (1), with decimal places being rounded up because index values are discrete. The computed q1 is the cutoff to divide HM into the elite and non-elite groups where g1∈{1,q1} and g2∈{(q1+1),HMS}.
  (1) $$q1=round{\left(0.25\times \left(HMS+1\right)\right)}^{th}\mathrm{term}.$$
  An extra parameter elit is included to allocate a proper weightage on the elite group. So, the selected new harmony has a higher probability to originate from the elite group. With a probability elit, a new harmony is selected from the elite group. If the selection is from the non-elite group, two harmonies will be picked. Then, the better one of the two will be the new harmony.
  By doing this, a better harmony is always selected. Note that a low quality harmony, when joining with other harmony or being adjusted, may also produce good harmony. Thus, elit cannot be too high to ensure a balance to seek from elite and non-elite group. The detailed selection process is illustrated in Algorithm 1.
**Algorithm 1**
selection( )
g1∈{1,q1}/*refer Equation (1)*/g2∈{(q1+1),HMS}/*refer Equation (1)*/
**if**(U(0,1)≤elit)
       $ind1=round(g1_{min}+U\left(0,1\right)\times \left(g1_{max}-g1_{min}\right))$
       ind=ind1
**else**
       $ind2=round(g2_{min}+U\left(0,1\right)\times \left(g2_{max}-g2_{min}\right))$
       $ind3=round(g2_{min}+U\left(0,1\right)\times \left(g2_{max}-g2_{min}\right))$
       **if**(ind2≤ind3)
              ind=ind2
       **else**
              ind=ind3
return ind
2. Dynamic HMCR and PAR with step function
  The numerical experiment demonstrates repeated trials with different combinations of HMCR and PAR for the hybrid HS models. The competitive results reported from the different combinations indicated that the recommended range by [34] (HMCR=[0.70,0.95] and PAR=[0.10,0.35]) is an appropriate range for both operators. Along with the elitism selection, it is important to ensure sufficient exploration and exploitation of the search process before reaching convergence. Thus, the HMCR and PAR is designated to be dynamic following an increasing and decreasing step function, respectively.
  The increasing and decreasing step function of HMCR and PAR cooperates with each other for a balance of exploration and exploitation. Initially, a lower HMCR provides an active global search to explore the search area and it works along with a higher PAR that provides an active local search to exploit the neighbourhood of the search area. Thus, HM consists of candidates scattering around the search area with its corresponding neighbourhood being well-exploited in the early stage of the search procedure. Following the step function, the global search exploration decreases and focuses in the search area stored in the HM, leading to the local exploitation to be focused in this specific search area. The dynamic settings of HMCR and PAR enable effective determination of the appropriate search area that lead to a more efficient convergence towards the final solutions.
  In utilizing the step function, several components, i.e., HMCR range, PAR range, HMCR increment, PAR decrement, and step size (step) have to be determined. Based on the numerical experiment conducted earlier, the range of the operators are set as the recommended range. The interval for increment and decrement of HMCR and PAR, respectively, is set at 0.05 as this small interval is sufficient to cover the whole range for these two operators. The step determines the number of iterations for HMCR and PAR to maintain before shifting to another value in the range until both operators reach a plateau. The setting of step depends on the search range size with a smaller step preferable as the main aim is to have faster convergence with active exploration and exploitation in the early stage of the search. Thus, step is set to enable both HMCR and PAR to reach a plateau within the first half of the total iterations. For the numerical experiment, MHS-SVM has step=10 while MHS-RF has step=5. The smaller step for MHS-RF is due to its smaller discrete search space than MHS-SVM with continuous search space.
3. Self-adjusted bw
  bw is an assistant tool for pitch adjustment and poses an effect on local exploitation. We suggest to replace the bw using a coefficient of variation (coef) (Equation 2) of the decision variable for every iteration, which will now be an auto-updated value in each iteration, thus enabling possible early convergence. This modification is only applicable for continuous decision variables as bw is not required for the adjustment of discrete decision variables.
  (2) $$\begin{array}{cc} s_{i} & =\frac{\sum_{j=1}^{HMS}\left(x_{i}^{j}-\bar{x_{i}}\right)}{HMS-1}\\ \bar{x_{i}} & =\frac{\sum_{j=1}^{HMS}x_{i}^{j}}{HMS}\\ coef_{i} & =s_{i}/\bar{x_{i}}\\ \forall i & =1,2,\ldots ,n. \end{array}$$
  This intuition comes from several past researches [35,36,37] that have proposed the improved HS with the bw modified. From these modifications, it is suggested that the dynamic bw should converge to smaller values as the iterations of the search process increases. Reference [35] recommended standard deviation (sd) as the appropriate replacement of bw. Reference [37] also utilized sd to replace bw, with an additional constant attached to control the local exploitation. Hence, using coef to substitute bw is an appropriate strategy because the division of sd with the mean is perceived as equivalent to the attached constant as in [37], yet has the benefit of being automatically updated in every iteration. Besides, coef can effectively scale the sd to ensure the search processes are maintained in an appropriate range. When the iterations increase, solutions in *HM* will converged, causing the coef to converge to smaller values.
4. Additional termination criteria
  The termination criteria used in this study are the maximum number of iterations (max_iter), convergence of *HM*, and non-improvement on the best solution for a fixed number of consecutive iterations (cons_no_imp). Since the previous three modifications open up the possibility for faster convergence, both criteria are included to avoid redundant iterations to save computational effort. MHS procedure will stop when any one of the criteria is met.

#### 2.3.2. Potential of MHS-SVM and MHS-RF

**Figure 3:**
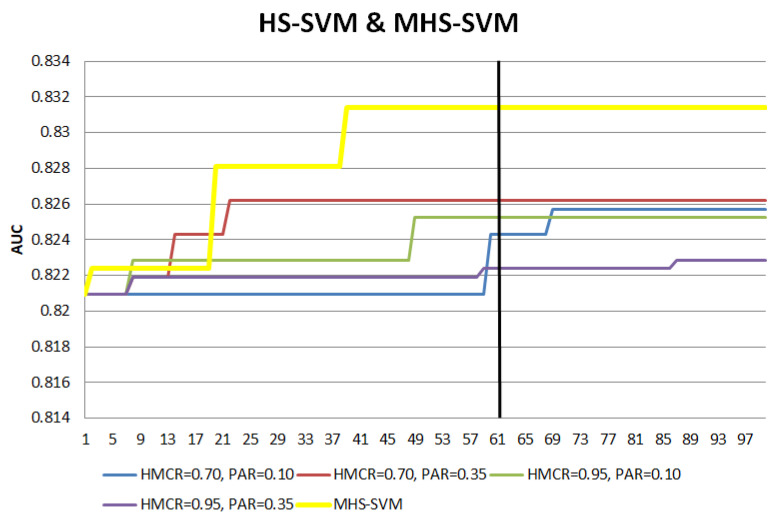
Search pattern comparison between HS-SVM with Modified HS (MHS-SVM).

**Figure 4:**
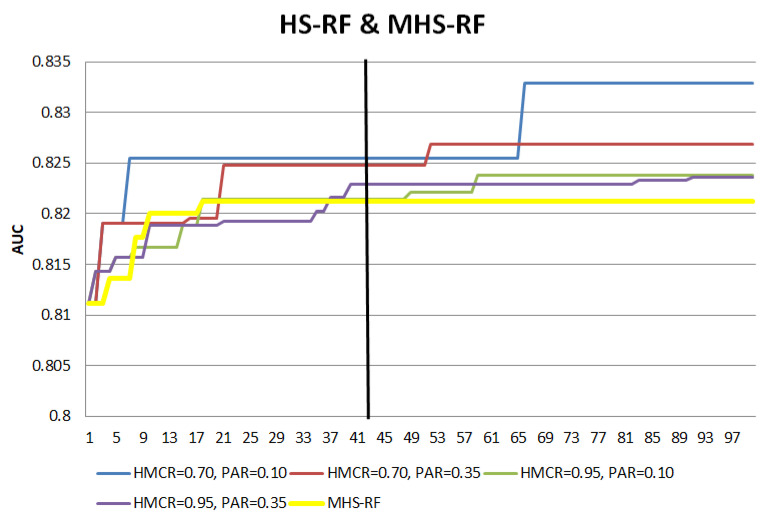
Search pattern comparison between HS-RF with MHS-RF).

The numerical experiment in Section 2.3.1 is repeated with the MHS hybrid models. The search patterns of the MHS hybrid models are compared with the HS hybrid models in Figure 3 and Figure 4 to illustrate the effect of the modifications.

For both MHS-SVM and MHS-RF, the modifications lead to earlier convergence, with the ‘a plateau’ pattern achieved much earlier compared to the HS hybrid models. The vertical lines in the figures mark the number of iterations required for the search process to end, while the AUC after the vertical lines is the result if the search process is allowed to run for the full number of iterations. With the MHS, the increment of AUC has shown a faster transition towards ‘a plateau’ with fewer numbers of iterations as compared to the HS hybrids, yet maintaining a comparable AUC performance (even with the other different settings of HS hybrid models). This indicates the MHS hybrid models have active exploration and exploitation in the earlier stages of the search, fulfilling the objectives of the MHS hybrid models to reach convergence with lesser iterations. This is the benefit of MHS hybrid models which can help by not needing to perform additional efforts for different HMCR and PAR combinations to check the model performance.

Table 3 compares the three different approaches, i.e., GS, HS, and MHS for the hyperparameter tuning task; in terms of measuring the AUC and the resulting computational time from the required number of iterations to end the search process. Overall, the HS hybrid models have potential in reducing computational effort especially when the hyperparameters are of continuous variables. The MHS hybrid models save up more computational efforts by reducing the number of iterations and the AUC reported are comparable to that of the GS approach and the standard HS.

## 3. Hybrid Models

HS and MHS act as the assistant tool to solve both hyperparameter tuning and model explainability tasks of SVM and RF models. All the proposed hybrid models are supported by machine learning theory as the underlying technique to carry out the final classification which consist of the supervised learning algorithms of SVM and RF, with HS and MHS hybridized together to improve model performances.

### 3.1. HS-SVM and MHS-SVM

SVM seeks for an optimal hyperplane with a maximum margin as the decision boundary to separate the two different classes. Given a training set with labelled instance pairs $\left(x_{i},y_{i}\right)$, where *i* is the number of instance i=1,2,3,…,m, $x_{i}\in R^{n}$ and $y_{i}\in \left\{-1,+1\right\}$. The decision boundary to separate two different classes in SVM is generally expressed as $w\cdot x_{i}+b=0$, which is the dot product between the weight vector *w* and data instances with the bias *b*.

The optimal hyperplane is found by solving the convex optimization problem as in Equation (3). The $\epsilon_{i}$ is the slack variable introduced to account for misclassification, with *C* as the accompanied penalty cost. To handle non-linearity, this study utilizes SVM with the Radial Basis Function (RBF) kernel, $\exp\{-\gamma ||{x_{i}-x_{j}||}^{2}\}$. Hence, the hyperparameters to be tuned for RBF-SVM are *C* and gamma.

(3) $$\begin{array}{cc} min\;\phi \left(w,b\right)=\frac{1}{2}{\left||w|\right|}^{2}+ & C\sum_{i=1}^{m}\epsilon_{i}\\ s.t.\;y_{i}\left(w\cdot x_{i}+b\right)\geq 1. \end{array}$$

HS-SVM and MHS-SVM are utilized to search for features subset and hyperparameters that can maximize the AUC of SVM. The full procedure of HS-SVM and MHS-SVM, as well as their differences are detailed as follows:

**Step** **1:** Define objective function and parameters of HS and MHS.
  The objective function is to maximize the AUC of the SVM classification function with three decision variables. The first decision variable, $x_{1}$ is a binary (0,1) string of length *a* (number of features in dataset), second ($x_{2}$) and third ($x_{3}$) decision variables correspond to the SVM hyperparameters search range $\log_{2}C=\left[-5,12\right]$ and $\log_{2}\gamma =\left[-12,5\right]$ [38], respectively. The detailed parameters settings are enclosed in Section 4.3.
**Step** **2:** Initialization of Harmony Memory
  Each harmony vector in HM has three decision variables. Every harmony vector is evaluated with the fitness function and sorted from the best to worst. Each decision variable is randomly initialized as in Equation (4). Both HS-SVM and MHS-SVM have the same HM.
  (4) $$\begin{array}{cc} x_{1}^{j} & =binary(0,1),\\ x_{2}^{j} & =min\left(x_{2}\right)+U\left(0,1\right)\times \left(max\left(x_{2}\right)-min\left(x_{2}\right)\right),\\ x_{3}^{j} & =min\left(x_{3}\right)+U\left(0,1\right)\times \left(max\left(x_{3}\right)-min\left(x_{3}\right)\right). \end{array}$$
**Step** **3:** Improvisation
  With probability HMCR, a new harmony is selected from the HM. The selected harmony is adjusted to the neighbouring values with a probability PAR. The two continuous variables ($x_{2},x_{3}$), the hyperparameters of SVM are adjusted to neighbouring values of width bw. With probability 1−HMCR, a new harmony vector is generated as in Equation (4).
  However, for the first decision variable, $x_{1}$ (which is the features), PAR operator acts as a flipping agent. When it is activated, the selected harmony will be flipped from 1 to 0 or vice versa. Note that not every feature is flipped as our aim is to adjust the harmony rather than randomize the harmony. The higher the fraction of features flipped, the more randomized is the harmony, causing it to resembles exploration instead of exploitation of the features, and altogether resulting in higher computational effort as the search process continuously explore other search space. Features fraction of more than half is considered as high randomization. On the other hand, the lower the fraction of features flipped, the lesser the harmony is being exploited. To ensure the functionality of the PAR operator as the exploitation tool, the midpoint between zero and half of the features to be flipped is selected. Thus, only a quarter of the features is flipped. This is controlled by flip, a random vector generating the feature numbers to be flipped.
  MHS-SVM will have the three modifications, i.e., dynamic HMCR and PAR following the step function in Equation (5), elitism selection, and replacement of bw with coef.
  (5) $$\left(HMCR_{iter},PAR_{iter}\right)=\left\{\begin{array}{cc} \left(0.70,0.35\right) & 1\leq iter<20\\ \left(0.75,0.30\right) & 20\leq iter<40\\ \left(0.80,0.25\right) & 40\leq iter<60\\ \left(0.85,0.20\right) & 60\leq iter<80\\ \left(0.90,0.15\right) & 80\leq iter<100\\ \left(0.95,0.10\right) & iter\geq 100. \end{array}\right.$$
  The improvisation procedure for HS-SVM and MHS-SVM are summarized in Algorithms 2 and 3, respectively.
**Step** **4:** Update HM by replacing the worst solution in HM with the new harmony if it has a better fitness value. This procedure is the same for HS-SVM and MHS-SVM.
**Step** **5:** Repeat Steps 3 and 4 until max_iter is reached for HS-SVM and for MHS-SVM when one of the two additional criteria, i.e., HM converges or no_cons_imp is reached.

**Algorithm 2****if**(U(0,1)≤HMCR)       ind=integer(U(0,1)×HMS)+1       $x_{1}^{\prime }=x_{1}^{ind}$       **if**
(U(0,1)≤PAR)               flip=rand(1:a,0.25×a)               $x_{1}^{\prime }=1-x_{1}^{ind}\left(flip\right)$**else**       $x_{1}^{\prime }=binary\left(0,1\right)$**if**(U(0,1)≤HMCR)       ind=integer(U(0,1)×HMS)+1       $x_{2}^{\prime }=x_{2}^{ind}$       **if**
(U(0,1)≤PAR)               $x_{2}^{\prime }=x_{2}^{ind}+U\left(-1,1\right)\times bw$**else**       $x_{2}^{\prime }=min\left(x_{2}\right)+U\left(0,1\right)\times \left(max\left(x_{2}\right)-min\left(x_{2}\right)\right)$**if**(U(0,1)≤HMCR)       ind=integer(U(0,1)×HMS)+1       $x_{3}^{\prime }=x_{3}^{ind}$       **if**
(U(0,1)≤PAR)               $x_{3}^{\prime }=x_{3}^{ind}+U\left(-1,1\right)\times bw$**else**       $x_{3}^{\prime }=min\left(x_{3}\right)+U\left(0,1\right)\times \left(max\left(x_{3}\right)-min\left(x_{3}\right)\right)$Note: a,b: Refer Equation (5)        c: Refer Algorithm 1        d: Refer Equation (2)


**Algorithm 3**

$HMCR=HMCR_{iter}$
^**a**^

$PAR=PAR_{iter}$
^**b**^

**if**
(U(0,1)≤HMCR)
       ind= selection ( )^**c**^       $x_{1}^{\prime }=x_{1}^{ind}$       **if**
(U(0,1)≤PAR)               flip=rand(1:a,0.25×a)               $x_{1}^{\prime }=1-x_{1}^{ind}\left(flip\right)$
**else**
       $x_{1}^{\prime }=binary\left(0,1\right)$
**if**
(U(0,1)≤HMCR)
       ind= selection ( )^**c**^       $x_{2}^{\prime }=x_{2}^{ind}$       **if**
(U(0,1)≤PAR)               $x_{2}^{\prime }=x_{2}^{ind}\pm CV_{2}$
^**d**^
**else**
       $x_{2}^{\prime }=min\left(x_{2}\right)+U\left(0,1\right)\times \left(max\left(x_{2}\right)-min\left(x_{2}\right)\right)$
**if**
(U(0,1)≤HMCR)
       ind= selection ( )^**c**^       $x_{3}^{\prime }=x_{3}^{ind}$       **if**
(U(0,1)≤PAR)               $x_{2}^{\prime }=x_{2}^{ind}\pm CV_{2}$
^**d**^
**else**
       $x_{3}^{\prime }=min\left(x_{3}\right)+U\left(0,1\right)\times \left(max\left(x_{3}\right)-min\left(x_{3}\right)\right)$Note: a,b: Refer Equation (5)        c: Refer Algorithm 1        d: Refer Equation (2)

### 3.2. HS-RF and MHS-RF

Random Forest is an ensemble model with a collection of decision trees using the bootstrap aggregation technique. Trees are grown using a binary splitting algorithm with Gini Impurity, $\mathrm{GI}=1-\sum_{i=1}^{k}p_{i}^{2}$ as the splitting criteria; where *i* is the number of classes and $p_{i}$ is the proportion of instances belonging to the respective class. During the tree growing process, to avoid correlations in between the trees, only a subset of the variables are required for splitting. The end result of the classification is based on the majority of votes from all the collected trees in the forest. The two hyperparameters to be tuned in RF are the number of trees (ntree) and number of variables available for splitting (mtry). HS-RF and MHS-RF are utilized to search for hyperparameters that can maximize the AUC of RF. The full procedure of HS-RF and MHS-RF, as well as their differences are detailed as follows:

**Step** **1:** Define objective function and parameters of HS and MHS.
  The objective function is the RF classification function with two decision variables that corresponds to the two hyperparameters, i.e., ntree and mtry. The search range for ntree is chosen to be discrete values of $x_{1}\in \left\{1,5\right\}$, where these values are then converted to the corresponding hundred. This search range is selected as it is often attempted by researchers. The search range of the second decision variable is discrete values of $x_{2}\in \left\{1,a\right\}$, where *a* is the total number of attributes available. This search range is chosen because the hyperparameter mtry is the random subset of variables from the total available attributes. The detailed parameters are enclosed in Section 4.3.
**Step** **2:** Initialization of Harmony Memory
  Each harmony vector in HM has two decision variables. Every harmony vector is evaluated with the fitness function and sorted from the best to worst. Since the decision variables to solve RF are discrete, the harmony vectors are sampled directly from the search range as in Step 1. Both HS-RF and MHS-RF have the same HM.
**Step** **3:** Improvisation
  With probability HMCR, a new harmony is selected from HM. Then the selected harmony is adjusted to the neighbouring values with probability PAR. As there are only discrete variables, the new harmony is adjusted directly to the left or right; bw is not required to adjust the new harmony. Hence only two modifications are involved in MHS-RF, i.e., dynamic HMCR and PAR following Equation (6) and the elitism selection.
  (6) $$\left(HMCR_{iter},PAR_{iter}\right)=\left\{\begin{array}{cc} \left(0.70,0.35\right) & 1\leq iter<5\\ \left(0.75,0.30\right) & 5\leq iter<10\\ \left(0.80,0.25\right) & 10\leq iter<15\\ \left(0.85,0.20\right) & 15\leq iter<20\\ \left(0.90,0.15\right) & 20\leq iter<25\\ \left(0.95,0.10\right) & iter\geq 25. \end{array}\right.$$
  The improvisation procedures for HS-RF and MHS-RF are summarized in Algorithms 4 and 5, respectively.

**Algorithm 4****for***i* in (1:2)       **if**
(U(0,1)≤HMCR)              ind=int(U(0,1)×HMS)+1              $x_{i}^{\prime }=x_{i}^{ind}$              **if**
(U(0,1)≤PAR)                      $x_{i}^{\prime }=x_{i}^{ind}\pm 1$       **else**              $x_{i}^{\prime }\in \left\{min\left(x_{i}\right),max\left(x_{i}\right)\right\}$Note: a,b: Refer Equation (6)        c: Refer Algorithm 1
**Algorithm 5**$HMCR=HMCR_{iter}$^**a**^$PAR=PAR_{iter}$^**b**^**for***i* in (1:2)       **if**
(U(0,1)≤HMCR)              ind= selection ( )^**c**^              $x_{i}^{\prime }=x_{i}^{ind}$              **if**
(U(0,1)≤PAR)                      $x_{i}^{\prime }=x_{i}^{ind}\pm 1$       **else**              $x_{i}^{\prime }\in \left\{min\left(x_{i}\right),max\left(x_{i}\right)\right\}$Note: a,b: Refer Equation (6)        c: Refer Algorithm 1

**Step** **4:** Update HM by evaluating and comparing the fitness function of the new harmony with the worst harmony in HM. Replace the worst harmony if the new harmony has better fitness value. This procedure is the same for both HS-RF and MHS-RF.
**Step** **5:** Repeat Steps 3 and 4 until max_iter is reached for HS-RF and for MHS-RF when one of the two additional criteria, i.e., HM converges or no_cons_imp is reached.

### 3.3. Parallel Computing

Both MHS-SVM and MHS-RF aim for quality results but faster convergence. Parallel computing with master-slave concept can be employed on the 10 independent tasks (from cross validation) to enhance the computational efficiency. Initially, the master generates sub-tasks via data preparation and splitting to be assigned to 10 slaves for independent and simultaneous execution. When done, each slave returns the required performance measures (refer Section 4.2 to compute the average. Algorithm 6 summarizes the parallel computation. Since the main aim is to save computational time, the same seeding is applied for both sequential and parallel execution to ensure identical model performance.
**Algorithm 6**Master: Data preparation and partitioning**do_parallel**       **for**
*i* in (1:10)               Slave: Step 1-5 of MHS-RFreturn AUC, ACC, ACC*Master: mean(AUC), mean (ACC), mean (ACC*)

## 4. Experimental Setup

### 4.1. Credit Datasets Preparation

The datasets used in the experiments are the German and Australian datasets which are publicly available at the UCI repository (https://archive.ics.uci.edu/). Additionally, a peer-to-peer lending dataset downloaded from the Lending Club (LC) website (https://www.lendingclub.com/info/download-data.action) is also included.

For the experiment, only the sample of 60-month-term of the year 2012 is taken because less attention was given on the 60-month-term loan in the past literature. To prepare the LC dataset, this experiment focuses only on loan status that are fully paid and charged off. Variables having all empty values or more than 5% missing values are removed, and variables with less than 1% of missing value have the whole instance being removed as it is only a small loss of information. Missing data is imputed with the mean for numerical and mode for categorical attributes, respectively. Table 4 gives a summary of the datasets. Attributes descriptions for German and Australian are available online while the brief descriptions of the LC attributes are shown in Table 5.

Numerical attributes are standardized by subtracting the column mean and dividing the standard deviation. Categorical attributes are converted to numerical attributes with the weight-of-evidence (WOE) transformation. 10-fold cross validation is applied on the datasets, and a validation set is prepared for the hyperparameter tuning procedure to avoid the overfitting problem. In the experiment, the German and Australian datasets are relatively small, thus the validation set is an inner 5-fold cross validation, whereas the relatively larger LC dataset has a holdout set as the validation set.

### 4.2. Performance Measures

This study utilizes both threshold-variant and threshold-invariant performance measures to evaluate the model performances. Accuracy (ACC) and the F1 score (F1) are reported at the default threshold at the cutoff probability of 0.5. ACC is the proportion of correctly classified instances in the data. For a more reliable estimate when there is class imbalance, F1 computes the harmonic mean of precision and recall is reported together for model evaluation. The threshold-invariant measure, AUC gives a better picture on the discriminating ability of a model across all possible thresholds. The Friedman test is conducted to test the significance of AUC between the compared models across the 10 test sets (from cross validation) for each dataset. The Wilcoxon signed rank test is applied if there is a significant difference reported from Friedman test.

This study assigns a positive sign to non-defaulting customers and a negative sign to defaulting customers. The Type I error represents acceptance of an actual defaulting customer whereas Type II error represents the rejection of an actual non-defaulting customer. Both types of errors result in a different extent of losses, depending on the financial environment of the institution. Hence, a different cutoff probability is usually adjusted to achieve a balance in between both types of errors. High sensitivity (SEN) and specificity (SPE) are equivalent to low Type II and Type I error, respectively. SEN and SPE are reported at the cutoff probability of 0.5 for a further discussion on the model performance in achieving a balance between both error types.

### 4.3. Models Setup

To assess the performance of the proposed models, Logistic Regression (LOGIT), Backward Stepwise Logistic Regression (STEP) and Linear Discriminant Analysis (LDA) are included for comparison as they are the standard statistical models in the credit scoring domain. The standard SVM and RF are tuned with the conventional GS, using the same grid points described in Section 2.2. Considering the extensive computational effort due to the cross validation setup explained in Section 4.1, only a coarse GS is conducted for SVM. Thus, there are five comparison models to be compared with the proposed models.

The detailed parameters settings of all hybrid models are shown in Table 6. HS and MHS hybrid models have their parameters set in the same way as in the numerical experiment described in Section 2.2 and Section 2.3. Hence, across the three datasets, HS hybrid models have different parameters settings whereas MHS hybrid models save up the effort of repeated trial-and-error due to the modification of dynamic HMCR and PAR step function. The MHS hybrid models step function for HMCR and PAR are setup as in Equations (5) and (6) for MHS-SVM and MHS-RF, respectively.

The proposed models are coded in R 3.5.1 and executed on a 2.70 GHz Intel(R) Core(TM) i7-7500 CPU with 4.00 GB RAM under Windows 10 operating system. For parallel computation, the parallel environment is initiated with the ‘doParallel’ library in R 3.2.5 and executed on a Linux based operating system using IBM system X360 M4 server with ten nodes of 2.0 GHz Intel Xeon 6C processors.

## 5. Results and Discussions

This section reports the experimental results obtained from the different credit scoring models across the three credit datasets based on model performances, model explainability and computational time.

### 5.1. Model Performances

Table 7 reports the models’ performances across the three datasets. For the German and Australian dataset, the AI models are competitive with the statistical models with only a slight performance difference within 2%. On the other hand, for the LC dataset, the AI models consistently outperformed the statistical models. This indicates the flexibility of the AI models to account for various data patterns. Focusing in the SVM and RF families, the proposed hybrid models have slightly improved AUC compared to the GS-tuned models. While the hybrid models do not show consistent improvement of ACC and F1 compared to GS approach, the performance difference has been maintained in a less than 1% margin. Hence, the reported performance measures have implied the hybrid models are very competitive when compared to the GS tuning method.

Based on the three performance measures, the SVM family models have a wider gap of performance difference than the RF family models. This is due to the functionality of the HS-SVM and MHS-SVM to conduct simultaneous feature selection with hyperparameter tuning at a smaller granularity than the GS approach. Therefore, HS-SVM and MHS-SVM will have a different input features subset with the GS-tuned SVM that utilized the full features. In addition, the ability of HS-SVM and MHS-SVM to directly search the continuous hyperparameters space also results in a slightly better performance than the coarse GS for SVM tuning in this experiment. HS-RF and MHS-RF report only very slight performance difference with GS tuned RF because no feature selection is conducted and the hyperparameters are discrete which results in the same search space for the three models.

ACC and F1 are threshold-variant performance measures that will change depending on the threshold settings. Hence, the Friedman statistical test is only conducted based on the threshold-invariant AUC, reported in the last row of Table 7, with the respective *p*-values enclosed in the parentheses. For the German and Australian datasets, despite the numerical differences, the Friedman tests do not show statistical significant differences between all the experimented models. For the LC dataset, the Friedman test shows statistically significant differences between the models. The corresponding post-hoc test with the *p*-values is tabulated in Table 8. The pairs that show significant differences at α=0.01 are marked in bold text.

The post-hoc Wilcoxon-signed ranked test shows statistically significant better AUC performance of both SVM and RF families than the statistical models. There is significant difference among the models in the SVM families, indicating the significant improvement of the proposed hybrid SVM models compared to the GS-tuned SVM. While there is no significant difference among the models from RF family, the slight difference in performance indicates that the proposed hybrid RF models are competitive to the GS-tuned RF. There is significant difference is reported between models from SVM and RF family, with RF family models having better performance.

Among the three performance measures, only AUC indicates consistent best performance from the RF family models and consistent improvements of proposed hybrid models compared to the GS tuning approach. Considering the ACC and F1, the performance ranking of the models are different across the three datasets. To consider all the performance measures together for a general overview evaluation of the models, each model is assigned an overall rank (ORank). In each dataset, the models are ranked based on their average rank computed across the three performance measures. The same reported performance will have a tied rank. All the rankings are tabulated in Table 9, with a lower value of ORank indicating better model performance.

According to the ORank, the RF family models take the best rank, followed by the SVM family models and lastly the statistical models. This order indicates the robustness of AI models compared to the statistical models. In addition, for both the RF and SVM family models, the proposed hybrid models always have a better ORank than the GS approach. Thus, HS is suitable to be hybridized with AI models for cautious hyperparameter tuning, and also feature selection; particularly SVM in this study. The hybrid models do not require specific settings of the granularity as in GS for continuous decision variables and at the same time is able to perform feature selection within the same time as a GS approach which only conducts hyperparameter tuning. The competitive performance of the proposed MHS hybrid models show that HS is very adaptable depending on user needs. MHS hybrid models effectively reduce computational effort yet at the same time maintains the quality of the solution (detailed discussions in Section 5.3).

Table 10 reports the model sensitivity and specificity across the three datasets. Instead of evaluating both measures separately, this study discusses both measures together as one single pair since a good model should not have dominance in only either one of it to achieve a balanced trade off between the two types of losses.

For the German and Australian dataset, both statistical and AI models have reported relatively similar sensitivity-specificity gaps, indicating a reasonable balance between Type II-Type I error. Models from the RF and SVM families have slight inclined priority towards a reduction of Type II error (due to higher sensitivity and lower specificity) in German and Australian datasets, respectively. For the LC dataset, the AI models report significant smaller sensitivity-specificity gaps compared to the statistical models. In contrast to statistical models that have extreme dominance in sensitivity, AI models have reported a reasonable balance between sensitivity and specificity, indicating that AI models have a better balance to reduce both Type I and Type II errors for the LC dataset. For the models of the RF family, the proposed hybrid models do not show consistent improvement across the three datasets compared to the GS-tuned RF but the reported performance have only a very slight difference, indicating the proposed models are competent. On the other hand, for the models from the SVM family, the proposed hybrid models have a slight improvement in the German and Australian dataset but significant improvement in the LC dataset compared to the GS-tuned SVM. This shows that HS and MHS have effectively improved the SVM performance by simultaneous feature selection and hyperparameter tuning.

Several recent literatures are outlined in Table 11 to highlight the main paradigm shift towards the usage of advanced AI techniques in credit scoring and the recent approach in credit scoring performance evaluation. Note that the abbreviations in Table 11 shall be referred to the original studies. Recent studies have much attention paid on advanced non-linear classifiers [29] and ensemble models, with tree-based ensembles [31,32] showing great potential because decision tree is perceived as the conventional classifier technique [33]. Besides, performance measures that is able to reflect the ability of the model in handling class imbalance is the recent trend where [33] have highlighted the usage of expected cost together with class imbalance for model evaluation and [29,30,31] have employed SEN, SPE and AUC for model evaluation. The summary from Table 11 implies the alignment of this study to fit with the recent paradigm, i.e., improvement of non-linear SVM and tree-based ensemble RF via a hybrid approach as well as model evaluation that takes into account for class imbalance via discussion on SEN and SPE.

Based on the summary in Table 11 and Table 12 compiled the studies that have experimented on the same dataset and utilized the same performance measures for results comparison with this study. Hence, only studies from [30,32] are included as comparison. Since [30] has proposed a novel approach in ranking the assessed models, only the top three models with the best rank are compiled. Due to the different assignment of positive sign for defaulting customers by [30], the true positive rate and true negative rate reported would be analogous to the SPE and SEN, respectively, in this study. For the study by [32], Australian dataset is the only data in common with our study with error rate as the single performance measure, thus ACC is computed from the error rate and reported in Table 12. Performance measures in bold texts indicate the best performance within that particular study.

The compiled results show that there is no obvious outperformance between the results reported by external studies with the proposed models. Across the two datasets over the four performance measures, the margin of difference have been maintained within 5% difference, which could not be considered as significant performance difference. This indirect comparison with external studies implies the competitiveness of the proposed models with the latest state-of-the-arts. It is worth to emphasize that model performance comparison with external studies would be difficult due to different experiment setup and varying proposed approaches to address different issues, the comparison in Table 12 aims to indicate the competency of the proposed models with current techniques instead of identifying a ‘winner’ among these experiments.

### 5.2. Model Explainability

For model explainability, the HS-SVM and MHS-SVM conduct feature selection and hyperparameter tuning simultaneously. The end user can then focus on the investigation of the reduced features subset. Table 13 reports the average number of reduced features across the 10-fold test sets. HS-SVM and MHS-SVM are compared only with STEP because STEP is the only model that conducted feature selection.

From Table 13, there is only a slight difference of the average number of reduced features between the three models for German and Australian datasets. However, for LC datasets, hybrid SVM models have more reduced features compared to STEP. For all the three datasets, the proposed hybrid SVM models have effectively reduced the features while maintaining a good performance as compared to the standard SVM that used the full features. This indicates that the proposed hybrid SVM models effectively reduced the original features but yet improved the standard SVM model.

For the RF models, this study recommends the use of the computed feature importance, i.e., the mean decrease in accuracy (mDA) and the mean decrease in Gini Impurity (mGI) for model explainability. Both mDA and mGI ranks the features from most to least important, thus providing the initial insight for the end user.

### 5.3. Computational Time

Table 14 reports the computational time of all the models utilized in Section 5.1, including the two parallel MHS hybrid models. Note that the performance measures for these two parallel hybrid models are identical, with only a difference in computational time, because of the same seeding applied.

Across the three datasets, the statistical models are very efficient, with only the STEP taking a longer time due to the feature selection process. The AI models are time-consuming due to the hyperparameter tuning process which is unavoidable as they are sensitive to the hyperparameters’ choice.

For both the SVM and RF families, similar computational effort can be perceived. In the experiments using the three datasets, the HS-hybrid models take the longest time, as compared to GS since it depends on the only termination criteria, max_iter in the HS procedure to ensure the search space is sufficiently explored. In contrast, MHS-hybridized models are able to search for comparable solutions as with the HS and GS, but in a much shorter time. This saves up to half of the computational effort. Together with parallel computing, the MHS hybrid models have more efficient computational power. Nonetheless, the development of the MHS hybrid models require experimentation and additional computational efforts. Between both the SVM and RF families, the SVM models are extremely time-consuming when the dataset contains more instances, i.e., the LC dataset. This is due to the training time complexity of SVM $O(n^{3})$.

Despite the benefit of being efficient without the need of hyperparameter tuning, the standard statistical models face limitations in dealing with more complex data patterns. In cases where the standard statistical models can no longer account for the data pattern, this results in poor performance. Further data transformation or interaction terms have to be included for the statistical models building procedure. This additional procedure may be another time-consuming process.

## 6. Conclusions and Future Directions

In this study, HS and MHS are hybridized with both SVM and RF, forming four new models. The newly proposed MHS is to ensure an effective yet efficient searching process. HS-SVM and MHS-SVM tune hyperparameters and reduces the features for model explanation while HS-RF and MHS-RF tune hyperparameters and utilize the two types of feature importance for model explainability. This allows flexibility in modeling while having high accuracy in the classification, comparative to traditional statistical modeling. In addition to this, the computational time is also competitive.

All the proposed models, HS-SVM, MHS-SVM, HS-RF, and MHS-RF, are competitive in the German and Australian datasets, and have great improvement over the standard statistical models in the LC dataset. All the HS and MHS hybrid models consistently reported higher AUC than the standard SVM and RF, implying the effectiveness of the proposed hybrid models to improve model discriminating ability. The proposed models only show slight improvement without significant difference in the German and Australian datasets, but the RF family models have shown statistically significant better results in LC dataset, with hybrid RF models reporting the best performance.

HS-SVM and MHS-SVM have effectively shrunk down the number of features, enabling end users to focus on the reduced features. HS-RF and MHS-RF are well-tuned, thus the computed feature importance are believed to be reliable in ranking the features. These strategies can be useful in providing initial insight for the end users.

In terms of computational effort, the standard statistical models are efficient as there is no hyperparameter tuning procedure required, but they fail to achieve good performance in the LC dataset. HS hybrid models are time consuming while MHS hybrid models have shown significant time saving. Along with parallel computing, the computational effort is further reduced. MHS hybrid models are very competitive compared to HS hybrid models, with the benefit of the computational efficiency being improved. In consideration of good discriminating ability, model explainability and computational efficiency, MHS-RF is the recommended alternative credit scoring model.

There are some possible future directions that can be pointed out. Instead of the time consuming standard SVM, other versions of SVM such as Least Squares SVM can be attempted to form a hybrid model. The model explainability approach in this study does not solve the black-box property of the AI models. Rules extraction can be incorporated to solve the black-box problem.

## Figures and Tables

**Table 1:** Performance of Harmony Search (HS)-Support Vector Machines (SVM) and HS-Random Forest (RF) across small perturbations of the operators.

		HS-SVM		HS-RF	
HMCR	PAR	AUC	Time (min)	AUC	Time (min)
0.70	0.10	0.8177	4.4481	0.8256	10.2604
0.70	0.15	0.8175	4.6211	0.8271	10.0100
0.70	0.20	0.8200	4.4203	0.8272	10.8761
0.70	0.25	0.8186	4.5008	0.8305	10.9019
0.70	0.30	0.8181	4.5614	0.8252	10.4817
0.70	0.35	0.8196	4.5122	0.8254	11.0123
0.75	0.10	0.8170	4.3773	0.8255	11.1008
0.75	0.15	0.8173	4.3323	0.8251	10.4266
0.75	0.20	0.8204	4.6357	0.8281	10.8729
0.75	0.25	0.8191	4.3702	0.8279	9.7096
0.75	0.30	0.8190	4.4640	0.8259	10.6458
0.75	0.35	0.8193	4.4810	0.8267	10.7759
0.80	0.10	0.8164	4.5614	0.8252	11.3625
0.80	0.15	0.8173	4.5198	0.8258	11.2265
0.80	0.20	0.8196	4.5138	0.8271	9.9028
0.80	0.25	0.8193	4.4698	0.8300	9.9752
0.80	0.30	0.8186	4.5357	0.8263	10.6068
0.80	0.35	0.8189	4.5541	0.8298	9.9372
0.85	0.10	0.8163	4.3997	0.8252	11.7332
0.85	0.15	0.8179	4.4258	0.8256	11.2218
0.85	0.20	0.8190	4.3716	0.8241	10.6202
0.85	0.25	0.8163	4.5028	0.8273	10.5372
0.85	0.30	0.8158	4.6122	0.8265	10.9275
0.85	0.35	0.8152	4.6928	0.8252	10.9275
0.90	0.10	0.8170	4.3648	0.8276	11.2554
0.90	0.15	0.8184	4.3502	0.8262	11.5087
0.90	0.20	0.8193	4.3950	0.8244	10.9275
0.90	0.25	0.8130	4.6232	0.8275	10.6972
0.90	0.30	0.8138	4.4245	0.8279	10.8565
0.90	0.35	0.8139	4.4260	0.8258	11.2159
0.95	0.10	0.8154	4.6132	0.8256	10.8480
0.95	0.15	0.8145	4.2717	0.8217	11.1239
0.95	0.20	0.8147	4.5164	0.8244	11.6584
0.95	0.25	0.8133	4.6013	0.8282	11.3777
0.95	0.30	0.8152	4.4070	0.8277	11.1307
0.95	0.35	0.8152	4.4630	0.8275	12.3353
	mean	0.8172	4.4817	0.8265	10.8754
	sd	0.0021	0.1000	0.0018	0.5785

**Table 2:** Comparison of Grid Search (GS) approach with HS hybrid models.

	AUC	Time (min)
GS-SVM	0.8078	23.9922
HS-SVM *	0.8172	4.4817
GS-RF	0.8214	9.0614
HS-RF *	0.8265	10.8754

* mean results from Table 1.

**Table 3:** Comparison of MHS hybrid models with HS Hybrids and GS Approach.

	AUC	Time (min)	Iterations
GS-SVM	0.8078	23.9922	614
HS-SVM *	0.8172	4.4817	100
MHS-SVM	0.8197	3.1502	71
GS-RF	0.8214	9.0614	100
HS-RF *	0.8265	10.8754	100
MHS-RF	0.8261	5.2008	49

* mean results from Table 1.

**Table 4:** Summary of benchmark datasets.

	Instances	Categorical	Numerical	Default Rate
German	1000	13	7	30%
Australian	690	8	6	44.45%
LC	9887	4	17	27.69%

**Table 5:** List of attributes in Lending Club (LC) dataset.

Attributes	Type	Attributes	Type
loan_amnt	Numerical	last_credit_pull_d ***	Numerical
emp_length *	Numerical	acc_now_delinq	Numerical
annual_inc	Numerical	chargeoff_within_12mths	Numerical
dti	Numerical	delinq_amnt	Numerical
delinq_2_yrs	Numerical	pub_rec_bankruptcies	Numerical
earliest_cr_line **	Numerical	tax_liens	Numerical
inq_last_6mths	Numerical	home_ownership	Categorical
open_acc	Numerical	verification_status	Categorical
pub_rec	Numerical	purpose	Categorical
revol_util	Numerical	initial_list_status	Categorical
total_acc	Numerical		

The full name of the attributes details can be found in the LCDataDictionary.xls file in the LC website. * Transformed from categorical. ** Transformed to how many years since first credit line opened. *** Transformed to how many months since LC pulled credit.

**Table 6:** Parameters settings of HS and MHS hybrid models for the three credit datasets.

	Parameters	German	Australian	Lending Club
HS-SVM	HMS	30	30	30
	HMCR	0.70	0.80	0.80
	PAR	0.30	0.10	0.30
	bw	0.10	0.10	0.10
	max_iter	1000	1000	1000
MHS-SVM	HMS	30		
	elit	0.70		
	HMCR	{0.70, 0.95}		
	PAR	{0.10, 0.35}		
	step	20		
	max_iter	1000		
	no_cons_imp	500		
HS-RF	HMS	10	10	10
	HMCR	0.70	0.70	0.80
	PAR	0.30	0.30	0.10
	max_iter	100	100	100
MHS-RF	HMS	10		
	elit	0.70		
	HMCR	{0.70, 0.95}		
	PAR	{0.10, 0.35}		
	step	5		
	max_iter	100		
	no_cons_imp	25		

**Table 7:** Model performances of the proposed hybrid models with GS-tuned AI models and statistical models.

	German			Australian			Lending Club		
	AUC	ACC	F1	AUC	ACC	F1	AUC	ACC	F1
LOGIT	0.7989	0.7590	0.8356	0.9308	0.8725	0.8462	0.6257	0.7239	0.8386
STEP	0.7999	0.7620	0.8378	0.9321	**0.8739**	0.8550	0.6245	0.7238	0.8387
LDA	0.8008	0.7470	0.8365	0.9286	0.8623	0.8473	0.6231	0.7238	0.8390
GS-SVM	0.8006	0.7440	0.8315	0.9292	0.8536	0.8486	0.7168	0.7236	0.8393
HS-SVM	0.8015	0.7620	0.8424	0.9313	0.8639	0.8579	0.8278	0.8251	0.8841
MHS-SVM	0.8051	0.7620	0.8403	0.9310	0.8565	0.8524	0.8267	0.8203	0.8800
GS-RF	0.7999	**0.7640**	0.8448	0.9354	0.8723	0.8598	0.8670	**0.8580**	**0.9068**
HS-RF	0.8044	**0.7640**	**0.8453**	**0.9366**	0.8738	**0.8614**	0.8674	0.8571	0.9063
MHS-RF	**0.8053**	0.7560	0.8410	0.9356	0.8695	0.8556	**0.8679**	0.8572	0.9064
	$\chi_{\mathrm{friedman}}^{2}=4.3685$, (0.8224)			$\chi_{\mathrm{friedman}}^{2}=5.8568$, (0.6633)			$\chi_{\mathrm{friedman}}^{2}=75.05$, (4.826e-13)		

**Table 8:** Post-hoc Wilcoxon-signed rank test for LC dataset of AUC performance.

	LOGIT	STEP	LDA	SVM	HS-SVM	MHS-SVM	RF	HS-RF	MHS-RF
LOGIT	-								
STEP	1.31 × 10^−1^	-							
LDA	8.40 × 10^−2^	3.23 × 10^−1^	-						
GS-SVM	**1.95 × 10^−3^**	**1.95 × 10^−3^**	**1.95 × 10^−3^**	-					
HS-SVM	**1.95 × 10^−3^**	**1.95 × 10^−3^**	**1.95 × 10^−3^**	**1.95 × 10^−3^**	-				
MHS-SVM	**1.95 × 10^−3^**	**1.95 × 10^−3^**	**1.95 × 10^−3^**	**1.95 × 10^−3^**	3.23 × 10^−1^	-			
GS-RF	**1.95 × 10^−3^**	**1.95 × 10^−3^**	**1.95 × 10^−3^**	**1.95 × 10^−3^**	**1.95 × 10^−3^**	**1.95 × 10^−3^**	-		
HS-RF	**1.95 × 10^−3^**	**1.95 × 10^−3^**	**1.95 × 10^−3^**	**1.95 × 10^−3^**	**1.95 × 10^−3^**	**1.95 × 10^−3^**	6.25 × 10^−1^	-	
MHS-RF	**1.95 × 10^−3^**	**1.95 × 10^−3^**	**1.95 × 10^−3^**	**1.95 × 10^−3^**	**1.95 × 10^−3^**	**1.95 × 10^−3^**	2.86 × 10^−1^	4.41 × 10^−1^	-

**Table 9:** Ranking of model performances across the three credit datasets.

	German				Australian				Lending Club				
	AUC	ACC	F1	Rank	AUC	ACC	F1	Rank	AUC	ACC	F1	Rank	ORank
LOGIT	9	6	8	7.7	7	3	9	6.3	7	7	9	7.7	7.3
STEP	7.5	4	6	5.8	4	1	5	3.3	8	8.5	8	8.17	5.9
LDA	5	8	7	6.7	9	7	8	8	9	8.5	7	8.17	7.7
SVM	6	9	9	8	8	9	7	8	6	6	6	6	6.4
HS-SVM	4	4	3	3.7	5	6	3	4.7	4	4	4	4	4.2
MHS-SVM	2	4	5	3.7	6	8	6	6.7	5	5	5	5	5.2
RF	7.5	1.5	2	3.7	3	4	2	3	3	3	1	2.3	3.1
HS-RF	3	1.5	1	**1.83**	**1**	2	1	**1.3**	2	2	3	2.3	**1.9**
MHS-RF	**1**	7	4	4	2	5	4	3.7	**1**	**1**	2	1.3	3.1

**Table 10:** Sensitivity and specificity analysis of the proposed hybrid models with GS-tuned AI models and statistical models.

	German		Australian		Lending Club	
	SEN	SPE	SEN	SPE	SEN	SPE
LOGIT	0.8757	0.4867	0.8678	0.8629	0.9919	0.0462
STEP	0.8786	0.4900	0.8848	0.8581	0.9930	0.0208
LDA	0.8743	0.4967	0.9165	0.8070	0.9952	0.0150
GS-SVM	0.9057	0.3667	0.9155	0.8043	0.9980	0.0069
HS-SVM	0.9086	0.4200	0.9185	0.8200	0.9229	0.5695
MHS-SVM	0.8957	0.4500	0.9252	0.8016	0.9130	0.5782
GS-RF	0.9187	0.4000	0.8699	0.8674	0.9555	0.6034
HS-RF	0.9229	0.3933	0.8634	0.8822	0.9564	0.5979
MHS-RF	0.9229	0.3667	0.8635	0.8746	0.9565	0.5979

**Table 11:** Recent literature studies in credit scoring domain.

Study	Proposed Approach	Classifier	Database	Performance Measures
[33]	Comparison of undersampling and oversampling to solve class imbalance	C4.5	4 UCI datasets* (A)	expected cost
[30]	3 MCDM methods to rank 9 techniques (Bayesian Network, Naive Bayes, SVM, LOGIT, k-nearest neighbour, C4.5, RIPPER, RBF network, ensemble)	Top three: LOGIT, Bayesian network, ensemble	2 UCI datasets* (G,A) and credit datasets representing 4 other countries	ACC, AUC, SEN, SPE, precision
[31]	Tree-based ensembles with synthetic features for features ranking and performance improvement	Extreme Gradient Boosting to learn ensemble of decision trees	EMIS database (Polish company)	AUC
[32]	Performance assessment of 5 tree-based ensemble models	AdaBoost, LogitBoost, RUSBoost, Subspace, Bagging	3 UCI datasets* (A)	error rate
[29]	Performance assessment of 4 neural network models	BPNN, PNN, RBFNN, GRNN with RT as benchmark	1 UCI dataset	ACC, SEN, SPE

* UCI dataset employed is the same as this study (G: German, A: Australian).

**Table 12:** Results comparison with external studies.

Data	Study	Model	ACC	AUC	SEN	SPE
Australian	[30]	LOGIT	**0.8623**	0.9313	0.8590	**0.8664**
		Bayesian Network	0.8522	0.9143	**0.8656**	0.7980
		Ensemble	0.8551	**0.9900**	0.8773	0.8274
	[32]	AdaBoost	0.8725	–	–	–
		LogitBoost	0.8696	–	–	–
		RUSBoost	0.8551	–	–	–
		Subspace	0.7667	–	–	–
		Bagging	**0.8939**	–	–	–
		HS-SVM	0.8639	0.9313	0.9185	0.8200
		MHS-SVM	0.8565	0.9310	**0.9252**	0.8016
		HS-RF	**0.8738**	**0.9366**	0.8634	**0.8822**
		MHS-RF	0.8695	0.9356	0.8635	0.8746
German	[30]	LOGIT	**0.7710**	0.7919	0.8900	**0.4933**
		Bayesian Network	0.7250	0.7410	0.8814	0.3600
		Ensemble	0.7620	**0.7980**	**0.8943**	0.4533
		HS-SVM	0.7620	0.8015	0.9086	0.4200
		MHS-SVM	0.7620	0.8051	0.8957	**0.4500**
		HS-RF	**0.7640**	0.8044	**0.9229**	0.3933
		MHS-RF	0.7560	**0.8053**	**0.9229**	0.3667

**Table 13:** Average number of reduced features.

	German	Australian	Lending Club
STEP	14.6	7	16.2
HS-SVM	14.9	8.4	5.8
MHS-SVM	13.9	9	6.1

**Table 14:** Computational time.

	German	Australian	Lending Club
LOGIT	0.3698 s	0.3624 s	5.8037 s
STEP	15.8467 s	0.3822 s	6.2699 min
LDA	0.5280 s	0.5339 s	3.0937 s
GS-SVM	49.3829 min	20.1442 min	3.6870 days
HS-SVM	101.4499 min	42.3236 min	4.652 days
MHS-SVM	36.8149 min	17.7540 min	1.4710 days
MHS-SVM (P)	5.764 min	3.405 min	9.854 h
GS-RF	49.4010 min	15.8474 min	1.9173 h
HS-RF	57.0272 min	30.0728 min	2.3945 h
MHS-RF	32.4922 min	12.2331 min	1.2369 h
MHS-RF (P)	5.5027 min	3.5496 min	12.5525 min

## Abbreviations

The following abbreviations are used in this manuscript:

ABC	Artificial Bee Colony
ACC	Accuracy
AF	Average Frequency
AI	Artificial Intelligence
AUC	Area Under Receiver Operating Characteristics
GA	Genetic Algorithm
GS	Grid Search
HM	Harmony Memory
HMCR	Harmony Memory Considering Rate
HMS	Harmony Memory Size
HS	Harmony Search
LDA	Linear Discriminant Analysis
LOGIT	Logistic Regression
MA	Metaheuristic Algorithm
mDA	mean Decrease Accuracy
mGI	mean decrease in Gini Impurity
MHS	Modified Harmony Search
PAR	Pitch-Adjusting Rate
PSO	Particle Swarm Optimization
RF	Random Forest
SEN	Sensitivity
SPE	Specificity
STEP	Backward Stepwise Logistic Regression
SVM	Support Vector Machine
